# Supplementary material for: Snow or rain? hybrid AI deciphers surface precipitation phase from satellite observations
Source: Nat Commun. 2026 Feb 16;17:2813. doi: 10.1038/s41467-026-69487-w (PMC13021960; doi:10.1038/s41467-026-69487-w)
Supplement: Supplementary file 1 — Supplementary Information [file 41467_2026_69487_MOESM1_ESM.pdf]

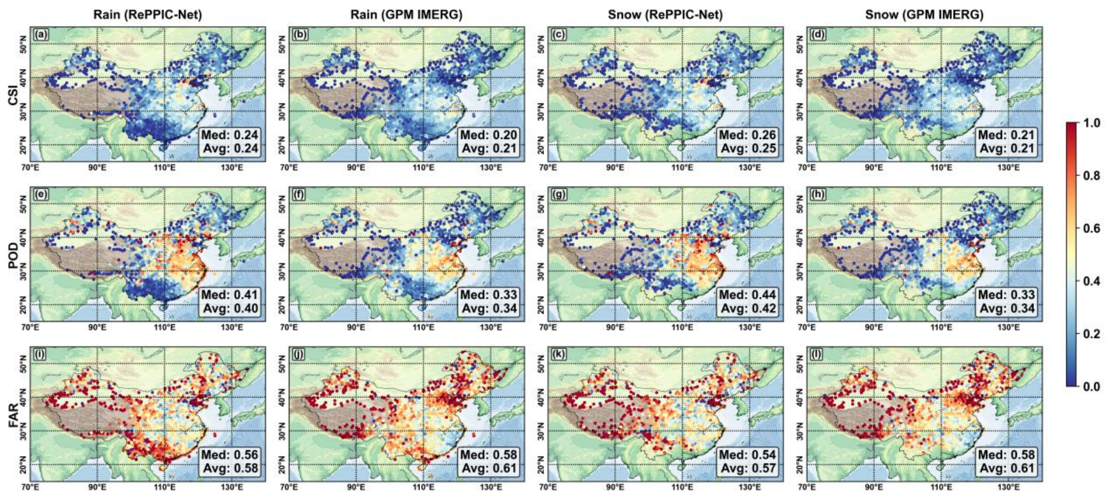

Supplementary Fig. 1| Evaluation of precipitation phase. Spatial distributions of the (a)-(d) Critical Success Index (CSI), (e)-(h) Probability of Detection (POD), and (i)-(l) False Alarm Ratio (FAR) for rain and snow from RePPIC-Net and GPM IMERG-Late.

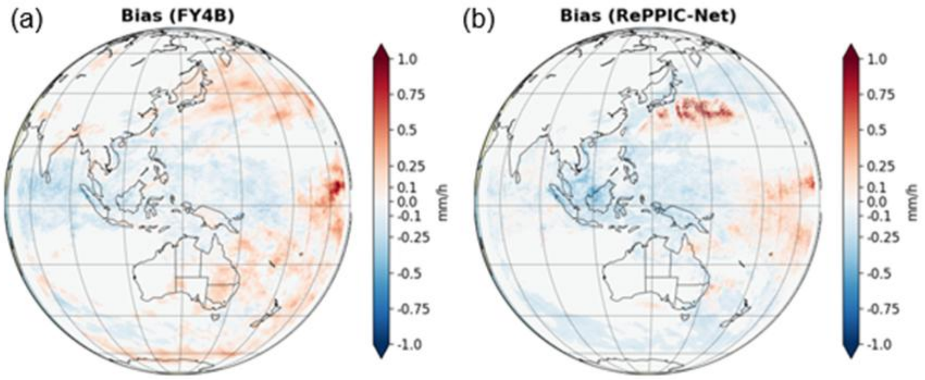

Supplementary Fig. 2| Systematic error distribution map of (a) FY-4B and (b) RePPIC-Net validated against GPM IMERG-Late.

Supplementary Table 1 Error analysis in the 12-hour FuXi forecasts and ERA5 reanalysis for meteorological variables used in precipitation retrieval models.

Variable	Bias	RMSE	CC
T500	-0.02	0.44	1.00
T700	0.02	0.57	1.00
T850	0.10	0.90	1.00
T925	0.09	1.01	1.00
R500	-0.35	8.49	0.96
R700	-0.58	7.58	0.96
R850	-0.81	6.79	0.96
R925	-0.61	6.80	0.96
U500	-0.04	1.42	0.99
U700	0.03	1.26	0.98
U850	0.03	1.07	0.98
U925	0.03	0.84	0.98
V500	0.04	1.40	0.99
V700	-0.08	1.26	0.98
V850	0.05	1.09	0.98
V925	0.06	0.94	0.98
TP	0.28	1.13	0.80

Supplementary Table 2 Evaluation of precipitation phase and detection using different meteorological field inputs.

		POD	FAR
Phase (rain)	ERA5	0.88	0.06
	FuXi (12h forecast)	0.91	0.08
Phase (snow)	ERA5	0.87	0.25
	FuXi (12h forecast)	0.83	0.19
Detection (rain)	ERA5	0.54	0.53
	FuXi (12h forecast)	0.53	0.45
Detection (snow)	ERA5	0.29	0.53
	FuXi (12h forecast)	0.27	0.41

Supplementary Table 3 Computational Complexity and Parameter Scale of Core Models.

Model	Task Type	FLOPs	Parameters
**UNet**	Precipitation Classification	664.011 M	7.711 M
**ResNet_UNet**	Precipitation Regression	3.958 G	13.154 M
**FuXi short model**	Atmospheric Field Prediction	—	1563.29 M
**DaYu-FY model**	Satellite BT Nowcasting	6.599 T	330.449 M

Supplementary Table 4 Full-disk evaluation based on GPM IMERG-Late.

	FY-4B QPE		RePPIC-Net	
	snow	rain	Snow	rain
RMSE	0.46	1.13	0.19	0.92
SSIM	0.97	0.91	0.98	0.93
